# Prevalence of maternal HIV infection and knowledge on mother–to–child transmission of HIV and its prevention among antenatal care attendees in a rural area in northwest Cameroon

**DOI:** 10.1371/journal.pone.0172102

**Published:** 2017-02-15

**Authors:** Carlson-Babila Sama, Vitalis F. Feteh, Maxime Tindong, John T. Tanyi, Nestor Mbinkar Bihle, Fru F. Angwafo

**Affiliations:** 1 Islamic Medicalized Health Centre, Babessi, Northwest Region, Cameroon; 2 Galactic Corps Research Group (GCRG), Buea, Southwest Region, Cameroon; 3 Mboppi Baptist Hospital Douala, Douala, Cameroon; 4 Health and Human Development (2HD) Research Group, Douala, Cameroon; 5 School of Public Health, Université Libre de Bruxelles, Bruxelles, Belgium; 6 Konye Sub–Divisional Hospital, Konye, Southwest Region, Cameroon; 7 Babessi Sub–Divisional Hospital, Babessi, Northwest Region, Cameroon; 8 Gynaeco-Obstetric and Paediatric Hospital, Yaoundé, and Department of Surgery, University Teaching Hospital, Yaoundé, Cameroon; University of North Carolina at Chapel Hill School of Dentistry, UNITED STATES

## Abstract

**Background:**

In 2010, an estimated 141 new HIV infections occurred per day in Cameroon and reports suggest an upsurge of these rates by 2020 if current trends continue. Mother—to—child transmission (MTCT) of HIV is a major public health challenge, and maternal knowledge on HIV transmission during pregnancy and its prevention is important in curtailing paediatric HIV acquisition.

**Objectives:**

We aimed at establishing the prevalence of maternal HIV infection as well as assessing knowledge on HIV, MTCT and prevention of MTCT (PMTCT) of HIV among pregnant women in a rural area of Cameroon.

**Methods:**

This study was conducted in two phases: a 29 month retrospective analysis of 1866 deliveries within three rural health facilities in the Babessi sub—division, Northwest Cameroon and a 1 month prospective phase wherein 150 consenting pregnant women attending antenatal care (ANC) at the study centres were consecutively recruited.

**Results:**

Overall, the prevalence of maternal HIV infection was 5.0% (100/2016). All (100%) of the interviewed pregnant women were aware of HIV infection and most (76.7%) had adequate knowledge on its routes of transmission. Meanwhile, only 79.3% (119/150) of them were aware of MTCT with slightly above a third (37.0%) having adequate knowledge on the periods of transmission. The proportions of women correctly stating: during pregnancy, during labour/delivery and during breastfeeding as possible periods of MTCT of HIV were 63.0%, 60.5% and 89.1% respectively. A majority (76.3%) of these women had inadequate knowledge on PMTCT of HIV.

**Conclusion:**

The overall prevalence of maternal HIV warrants strengthening of current intervention strategies including scaling—up of PMTCT measures. Among others, intensification of HIV—related ANC services to improve the pregnant women’s awareness and knowledge on MTCT and its prevention are vital steps in curbing the growing burden of paediatric HIV.

## Background

Over the last decades, the human immunodeficiency virus (HIV) has been one of the largest public health challenges especially in low—and middle—income countries (LMICs). Despite decades of sensitization and significant advances in its prevention and management, the pandemic continues to spread as an estimated 2 million global new HIV infections (220,000 occurred in children) were recorded in 2014, and there were about 36.9 million people living with HIV (PLWHIV) by the end of 2014 [1,2]. In contrast to earlier trends, HIV infection is now less prevalent in males with over half of infected adults being women [1,2]. As more women become infected, mother-to-child transmission (MTCT) of HIV continues to be a major challenge, especially in sub—Saharan Africa (SSA) where over two—thirds of the global burden of HIV/AIDS and its sequelae lie [1,2].

Similar to global trends, Cameroonian women are disproportionately affected as the prevalence of HIV among adult men and women aged 15–49 years was estimated at 2.9% and 5.6% respectively with an overall prevalence of 4.3%; the highest in West and Central Africa [3]. A serosurveillance survey showed an HIV prevalence of 7.6% among pregnant women [4]; and with an estimated 22.1% overall rate of MTCT in Cameroon [5], worsened by a very low antiretroviral therapy coverage (27.4%) [6], the number of new paediatric infections will continue to grow if appropriate measures are not taken. In 2010, reports from the National AIDS Control Committee (NACC) of Cameroon showed that about 7,300 babies were born HIV positive due to MTCT [7]; and without intervention, about half of these infected children will die before their second birthday [8]. Hence, targeting pregnant women is pivotal in the prevention of mother-to-child transmission (PMTCT) of HIV, as it will reduce the incidence of HIV infection, thus reducing HIV—related morbidity/mortality and importantly, relieving the associated significant financial burden especially in LMICs.

In an attempt to help curb the proportion of MTCT of HIV, the World Health Organization (WHO) called for the virtual elimination of paediatric HIV and recommended a four—pronged approach which includes: “primary prevention of HIV infection among women of childbearing age; preventing unintended pregnancies among women living with HIV; preventing HIV transmission from a woman living with HIV to her infant; and providing appropriate treatment, care and support to mothers living with HIV and their children and families” [1,9]. Evidently, maternal knowledge on MTCT plays a central role to the effective realisation of these recommendations, as preventive strategies are largely hinged thereon.

The purpose of this study was to establish the prevalence of maternal HIV infection as well as to assess the knowledge on HIV, MTCT and PMTCT of HIV among pregnant women attending antenatal care (ANC) in a rural sub—division in Northwest Cameroon.

## Methods

### Study design and setting

We carried out a descriptive and analytic cross—sectional study at three primary health facilities in Babessi sub—division, Ngoketunjia Division, Northwest Cameroon. This rural sub—division consists of four major chiefdoms namely Babessi, Babungo, Baba I and Bangolan. The population (over 50,000 inhabitants) are hugely reliant on subsistence and livestock farming [10]. The included health institutions were Babessi Sub—Divisional Hospital, Saint Monica Catholic Hospital and Islamic Medicalised Health Centre. These three institutions serve as referral centres and receive the bulk of deliveries and ANC attendees relative to all other health facilities within the sub—division put together. The study was conducted in two phases: a retrospective register analysis used to determine the prevalence of maternal HIV infection at delivery, and a prospective phase used to determine prevalence of HIV as well as to assess knowledge on HIV, MTCT and PMTCT of HIV among pregnant women.

### Participants, sampling and study procedure

For the retrospective phase, we reviewed all records of pregnant women who delivered during the period January 1^st^ 2014 and May 31^st^ 2016 at the study centres. Of the 1931 documented deliveries, 44 cases and 21 cases were excluded from the final analysis due to missing HIV status and indeterminate results respectively. Thus, 1866 cases were analysed. Data collection in this phase was done by the principal investigators.

The prospective phase was conducted over a period of 4 weeks (June 2016) using a pretested, structured, interviewer—administered questionnaire which was prepared after reviewing relevant literature [11–14]. Data collection was done during routine ANC days of the various study centres by regular clinic staff (state registered nurses) who had received prior training on data collection techniques. A convenience sampling technique was used whereby all pregnant women who presented for ANC services on the said days were consecutively approached for inclusion. Only consenting pregnant women were recruited to the survey, which was conducted prior to antenatal clinic lectures in private spaces at the study centres. No third party was allowed to stay with a participant during the interview. After the interview, participants were then screened for HIV infection (after a pre-test counselling) using Alere Determine^™^ HIV–1/2 rapid test (Abbott Laboratories, Abbott Park, Illinois, USA) in the laboratory of the health facilities by trained laboratory personnel. For all participants who tested positive to Determine^™^ HIV–1/2 rapid test, a second-line confirmatory test was done; SD Bioline HIV 1/2 3.0 (Standard Diagnostics, Inc.). All tests were carried out following the manufacturer’s guidelines. Post-test counselling was done by a trained counsellor, and confidential results were handed to the participant. The principal investigators supervised the data collection process in this phase and regularly checked the filled questionnaires for completeness and consistency. All interviewed participants were eventually restricted from participating in the study during their subsequent ANC visits.

### Measures

#### HIV sero—Status

Participants were considered HIV negative if Determine^™^ results were negative and only considered HIV positive if both Determine^™^ and Bioline^™^ tests were positive.

#### Knowledge on HIV transmission

Participants were considered aware of HIV when they report they had heard of HIV. Comprehensive knowledge about transmission of HIV was measured based on the following:

- If a pregnant woman correctly identified the four main modes of transmission of HIV (sexually, blood transfusion, infected sharps and MTCT)
- Rejecting common local misconceptions (i.e. HIV/AIDS can be transmitted through mosquito bites, supernatural means, and by physical contact e.g. hugging, handshakes)
- Being aware that a healthy—looking person can have HIV

Each correct response to the above questions was scored 1 point, otherwise 0. In total, the maximum obtainable score is 8 points. Participants with ≥6 correct responses were considered to have adequate knowledge on transmission of HIV; otherwise they were considered to have inadequate knowledge.

#### Knowledge on timing of MTCT of HIV

Participants were considered aware of the possibility of MTCT when they report HIV could be transmitted from an infected mother to her child. A pregnant woman was considered to have adequate knowledge on timing of MTCT of HIV if she correctly identified the three different periods (during pregnancy, during labour/delivery, and during breastfeeding) of MTCT of HIV; otherwise she was considered to have inadequate knowledge.

#### Knowledge on PMTCT of HIV

Participants were considered aware of the possibility of PMTCT when they report transmission of HIV from an infected mother to her child was preventable. Comprehensive knowledge was measured based on participants’ response to the following questions regarding ways of preventing MTCT of HIV

- Giving antiretroviral drugs to the mother
- Giving antiretroviral drugs to the newborn
- Delivery by caesarean section
- Avoidance of breastfeeding
- Avoidance of mixed feeding

Each correct response (Yes) to the above questions was scored 1 point, otherwise (No, I don’t know) was scored 0. In total, the maximum obtainable score is 5 points. Participants with ≥4 correct responses were considered to have adequate knowledge on prevention of MTCT of HIV; otherwise they were considered to have inadequate knowledge.

### Statistical analysis

Data was entered and analysed using the IBM-SPSS statistical software v.20 for Windows (SPSS Inc., Chicago, IL). Continuous variables have been reported as medians and 25^th^ and 75^th^ percentiles, and as means and standard deviations, while categorical variables were described through absolute (n) and relative (%) frequencies. Factors associated with adequate knowledge on MTCT and PMTCT of HIV were investigated using logistic regressions. A 2–sided p-value < 0.05 was considered statistically significant.

### Ethical consideration

Prior to commencement of study, ethical approval was obtained from the Ethical Committee of the Babessi sub—divisional council. Also, administrative clearance was obtained from the heads of the institutions involved. After explaining the study objectives and procedure in details, all enrolled participants in the prospective phase signed a consent form prior to recruitment. For participants younger than 18years, a written informed consent was obtained from the next of kin in accordance with the guidelines of the Ethical Committee. Confidentiality was maintained via coding of questionnaires in both the retrospective and prospective phases.

## Results

### Prevalence of maternal HIV infection

Of the 1866 cases analysed in the retrospective phase, the prevalence of maternal HIV infection at delivery was 4.9% (92/1866). Prospectively, a total of 156 pregnant women presented to the clinics and were approached for inclusion, among which 150 consented to the study (response ratio: 96.2%). Eight of these participants tested positive to both the Determine^™^ and Bioline^™^ tests, giving a prevalence of HIV of 5.3% (8/150). Overall, the prevalence of HIV infection was estimated at 5.0% (100/2016).

### Socio—demographic characteristics of antenatal care attending women

The mean age of the study participants was 25.4 ± 6.5 years. Teenagers (14–19 years) constituted 18.7% (28/150) of the study population. A vast majority (83.3%) of the study population were married amongst which about a third (30.4%) were married into polygamous homes. Over half (56.7%) of the participants were farmers and only a minority (4%) had attained a tertiary level of education (Table 1).

**Table 1:** Socio—demographic characteristics of antenatal care attending women, Babessi sub—division, 2016.

Variable	Frequency (n)	Percentage (%)
**Age**		
14–25	82	54.7
26–35	56	37.3
36–42	12	8.0
**Marital status**		
Married	125	83.3
Single	18	12.0
Divorced	2	1.3
Widowed	2	1.3
Cohabiting	3	2.0
Type marriage*		
Monogamy	87	69.6
Polygamy	38	30.4
**Religion**		
Christian	98	65.3
Muslim	46	30.7
Traditionalist	1	0.7
Others	5	3.3
**Educational level**		
None	8	5.4
Primary	62	41.3
Secondary	74	49.3
Tertiary	6	4.0
**Occupation**		
Housewife	17	11.3
Farmer	85	56.7
Jobless	1	0.7
Student	18	12.0
Self—employed	17	11.3
Trader	10	6.7
Government work	2	1.3

*for married participants

### Obstetric characteristics of antenatal care attending women

The mean gestational age at booking visit was 20.5 ± 6.3 weeks with majority (73.3%) of the participants booking in the second trimester of their pregnancies. A fifth (19.3%) were currently at their first ANC visit and over half (56%) were at the third trimester of their pregnancy. Most (62.7%) were between the second and fourth ANC visits with an overall mean attendance of 3 ± 1.7 visits (Table 2).

**Table 2:** Obstetric characteristics of antenatal care attending women, Babessi sub—division, 2016.

Variable	Categories	Frequency (n)	Percentage (%)
**Gravidity**	1	33	22
Mean: 3.7 ± 2.5	2–4	65	43.3
Range: 1–14	≥ 5	52	34.7
**Parity**	0	38	25.3
Mean: 2.4 ± 2.2	1–4	83	55.3
Range: 0–9	≥ 5	29	19.4
**Gestation at Booking**	1^st^ trimester	23	15.3
Mean: 20.5 ± 6.3	2^nd^ trimester	110	73.3
Range: 8–36	3^rd^ trimester	17	11.3
**Gestational age of current pregnancy**	0–12 weeks	0	0
Mean: 30.3 ± 7.2	13–28 weeks	58	38.7
Range: 15–44	29–40 weeks	84	56.0
	≥ 41 weeks	3	2.0
**Number of ANC attendance for current pregnancy**	1^st^	29	19.3
Mean: 3.0 ± 1.7	2^nd^– 4^th^	94	62.7
Range: 1–9	≥ 5^th^	27	18.0

### Awareness and knowledge on HIV, MTCT and PMTCT of HIV among antenatal care attending women

**Fig 1:**
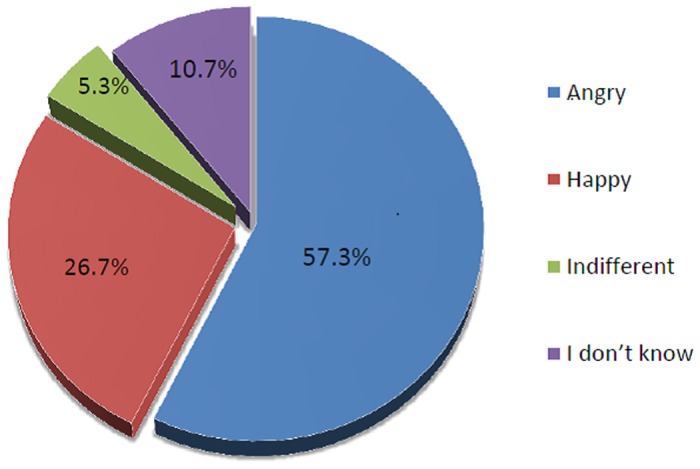
Perceived reaction of male partners if their antenatal care attending women underwent HIV testing without their permission.

All participants (100%) stated they were aware of the existence of HIV with most (55.4%) being aware of it for over 10 years. The overall mean HIV knowledge score was 6.3 ± 1.4 with a median of 6.0 (25th– 75^th^ percentiles: 6.0–7.0). Over three quarters (76.7%) of the participants had adequate knowledge on HIV. Ninety—four (64.8%) of the participants knew the HIV status of their partners while 99 (68.3%) reported they needed permission from their partners before undergoing the HIV test. Over half (57.3%) of the respondents stated their partners would be “angry” if they found out they did the HIV test without their consent (Fig 1).

Of all participants, 119 (79.3%) were aware of the possibility of MTCT of HIV. Breastfeeding was the most common (89.1%) period of transmission cited by the mothers (Table 3). Overall, the mean knowledge score on MTCT of HIV was 2.1 ± 0.8 with a median of 2.0 (25th– 75^th^ percentiles: 1.0–3.0). Slightly above a third (37.0%) had adequate knowledge on the periods of MTCT of HIV.

**Table 3:** Awareness and knowledge on MTCT and PMTCT of HIV among antenatal care attending women, Babessi sub—division, 2016.

Variable		Frequency (n)	Percentage (%)
**Awareness on MTCT (N = 150)**	Aware	119	79.3
	Unaware	31	20.7
**Reported period of MTCT (n = 119)**	During pregnancy	75	63.0
	During labour/delivery	72	60.5
	During breastfeeding	106	89.1
**Knowledge on MTCT**	Adequate	44	37.0
	Inadequate	75	63.0
**Awareness of PMTCT (n = 119)**	Aware	118	99.2
	Unaware	1	0.8
**Reported methods of PMTCT (n = 118)**	ARV to mother	113	95.8
	ARV to newborn	92	78.0
	Caesarean delivery	43	36.4
	Avoid breast feeding	96	81.4
	Avoid mixed feeding	28	23.7
**Knowledge on PMTCT**	Adequate	28	23.7
	Inadequate	90	76.3

**Fig 2:**
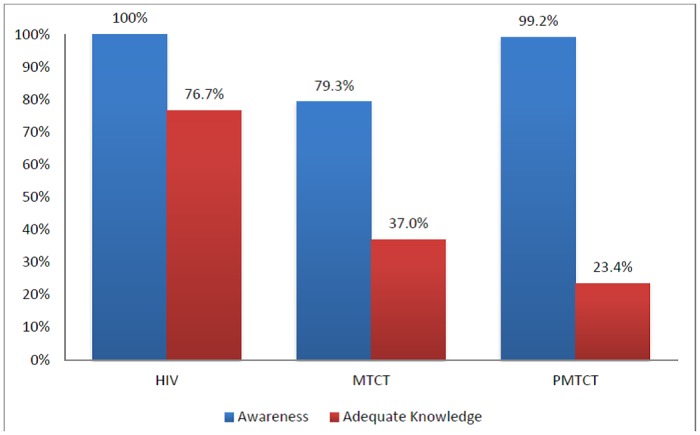
Awareness and knowledge of HIV, MTCT and PMTCT of HIV among antenatal care attending women, Babessi sub—division, 2016.

Almost all [99.2% (118/119)] of the participants who were aware of MTCT of HIV stated it can be prevented. Amongst others, giving ARVs to an infected mother (95.8%) was the most known preventive method while avoidance of mixed feeding (23.7%) was the least known (Table 3). The mean knowledge score on PMTCT of HIV among respondents was 2.9 ± 1.1 with a median of 3.0 (25th– 75^th^ percentiles: 2.0–3.0). Less than a quarter (23.7%) had adequate knowledge on preventive methods. Fig 2 summarises the awareness and knowledge of HIV, MTCT and PMTCT of HIV among the study participants.

### Factors associated with adequate knowledge on MTCT and PMTCT of HIV

Marital status, age, level of education, income, gravidity, HIV status, ANC attendance, and knowledge on HIV were tested for association with adequate knowledge on MTCT and PMTCT. None of the tested variables was found to be associated with adequate knowledge of MTCT (Table 4) and PMTCT (Table 5) of HIV in univariable logistic regression models.

**Table 4:** Unadjusted correlates of adequate knowledge on MTCT of HIV among antenatal care attending women, Babessi sub—division, 2016.

Variables	Categories	Frequency n (%)	Odds ratio (95% CI)	*p*–value
**Age**	>25	68 (45.3)	1.42 (0.68–2.98)	0.35
	≤ 25	82 (54.7)	1	
Partner*	Yes	128 (85.3)	1.39 (0.51–3.77)	0.51
	No	22 (14.7)	1	
**Religion**	Christian	98 (68.1)	1.49 (0.63–3.51)	0.36
	Muslim	46 (31.9)	1	
**Education**	Primary and below	70 (46.7)	1	
	Secondary and above	80 (53.3)	0.77 (0.37–1.61)	0.49
**Occupation**^#^	Income	114 (76.0)	1.71 (0.69–4.24)	0.25
	No Income	36 (24.0)	1	
**Gravidity**^&^	Primigravida	33 (22.0)	1	
	Multigravida	65 (3.3)	0.94 (0.35–2.49)	0.89
	Grand Multigravida	52 (34.7)	1.92 (0.71–5.21)	0.20
**HIV status**	Positive	8 (5.3)	1.08 (0.25–4.76)	0.92
	Negative	142 (94.6)	1	
**Number of ANC attendance**	1	29 (19.3)	1	
	2–4	94 (62.7)	0.96 (0.38–2.47)	0.94
	≥ 5	27 (18.0)	1.35 (0.43–4.24)	0.61
**Gestational age at booking**	1^st^ trimester	23 (15.3)	1	
	2^nd^ trimester	110 (73.3)	1.4 (0.30–6.53)	0.67
	3^rd^ trimester	17 (11.3)	0.93 (0.25–3.43)	0.93
**Knowledge on HIV**	Adequate	115 (76.7)	0.69 (0.29–1.68)	0.42
	Inadequate	35 (23.3)	1	

*Partner = (married + cohabiting) and no partner = (single + divorced + widow)

^#^Income = (farmer, self—employed, trader, government work) and no income = (housewife + jobless + student)

^&^Gravidity: Primigravida = 1 delivery; Multigravida = 2–4 deliveries; Grand multigravida = ≥5 deliveries

**Table 5:** Unadjusted correlates of adequate knowledge on PMTCT of HIV among antenatal care attending women, Babessi sub—division, 2016.

Variables	Categories	Frequency n (%)	Odds ratio (95% CI)	*p*–value
**Age**	>25	68 (45.3)	1.61 (0.70–3.71)	0.27
	≤ 25	82 (54.7)	1	
Partner*	Yes	128 (85.3)	1.17 (0.36–3.86)	0.79
	No	22 (14.7)	1	
**Religion**	Christian	98 (68.1)	1.42 (0.54–3.71)	0.48
	Muslim	46 (31.9)	1	
**Education**	Primary and below	70 (46.7)	1	
	Secondary and above	80 (53.3)	1.10 (0.47–2.55)	0.83
**Occupation**^#^	Income	114 (76.0)	1.06 (0.40–2.80)	0.90
	No Income	36 (24.0)	1	
**Gravidity**^&^	Primigravida	33 (22.0)	1	
	Multigravida	65 (3.3)	2.24 (0.67–7.52)	0.19
	Grand Multigravida	52 (34.7)	1.67 (0.46–6.10)	0.44
**HIV status**	Positive	8 (5.3)	1.32 (0.24–7.19)	0.75
	Negative	142 (94.6)	1	
**Number of ANC attendance**	1	29 (19.3)	1	
	2–4	94 (62.7)	1.0 (0.35–2.89)	1.0
	≥ 5	27 (18.0)	0.60 (0.15–2.47)	0.48
**Gestational age at booking**	1^st^ trimester	23 (15.3)	1	
	2^nd^ trimester	110 (73.3)	1.97 (00.53–7.33)	0.31
	3^rd^ trimester	17 (11.3)	2.25 (0.37–13.67)	0.38
**Knowledge on HIV**	Adequate	115 (76.7)	1.80 (0.56–5.75)	0.32
	Inadequate	35 (23.3)	1	

*Partner = (married + cohabiting) and no partner = (single + divorced + widow)

^#^Income = (farmer, self—employed, trader, government work) and no income = (housewife + jobless + student)

^&^Gravidity: Primigravida = 1 delivery; Multigravida = 2–4 deliveries; Grand multigravida = ≥5 deliveries

## Discussion

Our study has shown a high prevalence of maternal HIV infection and that pregnant women in this rural setting are aware of HIV with adequate knowledge on its transmission. However, significant awareness and knowledge deficits on mother—to—child transmission of HIV and its prevention were noted among these ANC attendees. These findings are in line with studies conducted in similar low—resource settings [14–16].

In Cameroon, there is a regional variation in the reported prevalence of HIV among the general population with the Northwest Region (6.3%) being second only to the South Region (7.2%) [3]. A national estimate of the prevalence of HIV among pregnant women stood at 5% [3], a finding mirrored by our results. Our prevalence is similar to the national prevalence (5.3%) among ANC attending women in Tanzania [17] but it is lower than the 10.3% observed in Fako Division, Southwest Cameroon [18]; in line with national statistics which showed a higher prevalence of HIV infection among urban dwellers compared to those living in rural areas [3]. The observed high prevalence of HIV infection among pregnant women in this study together with the reported high rates of HIV seroconversion during pregnancy and rate of MTCT in Cameroon [5,18] synergistically make up a potential risk for new paediatric HIV infections.

Though all pregnant women were aware of HIV with majority (76.7%) having adequate knowledge on its methods of transmission, up to a fifth (20.7%) were not aware of the possibility of MTCT. This finding concurs to reports of Lamina et al. in Nigeria [16] and Awungafac and colleagues [19] in a community—based study in the Tiko Health District, Southwest Cameroon where only three—quarters of the women had ever heard of MTCT of HIV. Furthermore, only 37.0% of women who were aware of MTCT of HIV in this study had adequate knowledge on possible transmission periods. This observation is at variance to findings in more urban settings in Cameroon where higher awareness/knowledge levels were noted [18,20]; thus suggesting demographic influence on maternal knowledge levels which goes in line with reports across varied African settings where it was observed that women in urban areas were more likely to have adequate knowledge on MTCT of HIV than those residing in rural settings [11,13,14,21]. This disparity maybe accounted for, in part by the fact that women in urban areas attend ANC in health facilities with a relatively better staff strength and also have more access to information via widespread availability of varying media streams. The significant knowledge gap between HIV and MTCT of HIV observed in this study also puts into question the quality of ANC services offered to these women and if the information is adequately comprehensible.

As noted in this study, breastfeeding was the most common known period of MTCT of HIV among women, a finding which coincides with reports from some authors [12,19], while others have observed women to be more aware of transmissions during pregnancy [11,16] and during labour/delivery [13,14,20]. These variations across different settings suggests there is a general lack of adequate knowledge on the timing of MTCT of HIV thus warranting the need for more objective sensitisation campaigns. Such targeted campaigns should aim at raising knowledge levels not only among pregnant women, but the population at large which will eventually help curb the growing burden of HIV.

The high awareness on avoidance of breastfeeding (81.4%) as a method of PMTCT of HIV by our respondents may be a reflection of the high awareness of breastfeeding as a possible period of MTCT of HIV (89.1%). Similar to findings from Uganda [12], most of our participants recognized the protective effects of antiretroviral drugs (ARV) in preventing MTCT of HIV. This finding somewhat suggests a positive attitude towards uptake of ARVs by infected mothers in a bid to reduce the risk of infecting their babies. Further studies are however recommended to elucidate the relationship between knowledge and uptake of ARVs by infected mothers.

Mixed feeding, seemingly still a common practice in rural milieus, is an important independent predictor of MTCT of HIV with reported odds as high as 42 times in mixed—fed infants compared to exclusively breastfed infants [22]. However, less than a quarter (23.7%) of our participants recognised this significant risk factor for MTCT of HIV. As evidenced in Ethiopia [22–24], this may be one of the reasons why the number of new paediatric infections keeps rising given that almost half of the Cameroonian population is rural [25]. Overall, over three quarters (76.3%) of our participants had inadequate knowledge on PMTCT measures which further suggest significant knowledge disparities between urban and rural dwellers as better knowledge levels have been reported in more urbanised settings in Cameroon [18,20].

Given that over 90% of the global new paediatric HIV infections recorded in 2014 resulted from MTCT [1,2], the low levels of adequate knowledge on MTCT/PMTCT of HIV observed in this study creates an imperative for the designing of targeted strategies to raise awareness and encourage uptake of preventive methods by infected mothers. However, preventive methods such as avoidance of breastfeeding may not be practicable in low—resource settings especially in sub—Saharan Africa due to intrinsic sociocultural values and economic constraints as substitute feeding may not be acceptable, affordable, safe and sustainable. Similarly, in such settings, elective caesarean sections may lead to an unbearable financial burden and equally carry negative cultural perceptions as inability to deliver via the vaginal route is often regarded as a reproductive failure [26]. Thus, intervention strategies should be geared at raising awareness, tailored maternal education and scaling—up ARV coverage as well as encouraging safe breastfeeding practices.

Amidst the limitations, our study being cross—sectional in nature implies causality cannot be inferred from our findings. The small sample size in the prospective phase is also a potential limitation as it may render estimates as well as associations unstable and therefore undetected. Furthermore, our findings are confined to a group of pregnant ANC attendees in a rural sub—division thus affecting the generalizability of results as it does not necessarily reflect knowledge levels in other rural as well as urban settings within the nation. However, being a pioneer study within the region, the attention our study deserves from health policy makers cannot be overemphasized, with consequent urgent need to improve awareness/knowledge levels.

## Conclusion

About 1 in 20 pregnant women in Babessi sub—division is HIV positive. Whilst being fully aware of HIV with adequate knowledge on its transmission routes, awareness and knowledge levels on timing of mother—to—child transmission of HIV and its prevention among ANC attendees are sub—optimal. This therefore reiterates the invaluable need to strengthen existing ANC modules as well as devising alternative strategies aimed at improving maternal knowledge on MTCT of HIV and scaling—up of PMTCT measures.

## Supporting information

S1 File STROBE checklist. (DOC)

S2 File Data collection sheet. (DOCX)

S3 File Dataset. (SAV)
