# Supplementary material for: Prevalence of maternal HIV infection and knowledge on mother–to–child transmission of HIV and its prevention among antenatal care attendees in a rural area in northwest Cameroon
Source: PLoS One. 2017 Feb 15;12(2):e0172102. doi: 10.1371/journal.pone.0172102 (PMC5310783; doi:10.1371/journal.pone.0172102)
Supplement: S1 File — (DOC) [file pone.0172102.s001.doc]

STROBE Statement—Checklist of items that should be included in reports of ***cross-sectional studies***

|  | Item No | Recommendation |
| --- | --- | --- |
| **Title and abstract** | *1* | *(a) Indicate the study’s design with a commonly used term in the title or the abstract*  This has been done in the abstract. This was a cross-sectional study ( page 2) |
|  |  | *(b) Provide in the abstract an informative and balanced summary of what was done and what was found*  This has been done with explanation of main outcomes and principal results (page 2) |
| Introduction | | |
| Background/rationale | *2* | *Explain the scientific background and rationale for the investigation being reported*  This has been done. See page 3 – 4. |
| Objectives | *3* | *State specific objectives, including any prespecified hypotheses*  This has been done. See first paragraph page 3 – 4. |
| Methods | | |
| Study design | *4* | *Present key elements of study design early in the paper*  This has been done in the subsection “study design and setting” (page 4) |
| Setting | *5* | *Describe the setting, locations, and relevant dates, including periods of recruitment, exposure, follow-up, and data collection*  All these data has been included in the methodology section (page 4) |
| Participants | *6* | *(a) Give the eligibility criteria, and the sources and methods of selection of participants*  All these data has been included in subsection “Participants, sampling and study procedure” (page 4-5) |
| Variables | *7* | *Clearly define all outcomes, exposures, predictors, potential confounders, and effect modifiers. Give diagnostic criteria, if applicable*  This has been done in the Methods (page 4 – 6) |
| Data sources/ measurement | *8**** | *For each variable of interest, give sources of data and details of methods of assessment (measurement). Describe comparability of assessment methods if there is more than one group*  All these data has been included in the methodology (page 4 - 5) |
| Bias | *9* | *Describe any efforts to address potential sources of bias* |
| Study size | *10* | *Explain how the study size was arrived at*  This data has been included in subsection “Participants, sampling and study procedure” (page 4) |
| Quantitative variables | *11* | *Explain how quantitative variables were handled in the analyses. If applicable, describe which groupings were chosen and why*  How quantitative variables were handled is explained in the subsection “Statistical analysis”. (page 6) |
| Statistical methods | *12* | *(a) Describe all statistical methods, including those used to control for confounding* |
|  |  | *(b) Describe any methods used to examine subgroups and interactions* |
|  |  | *(c) Explain how missing data were addressed* |
|  |  | *(d) If applicable, describe analytical methods taking account of sampling strategy* |
|  |  | *(e) Describe any sensitivity analyses*  All information has been provided in subsection “Statistical analysis”. (page 6) |
| Results | | |
| Participants | *13**** | *(a) Report numbers of individuals at each stage of study—eg numbers potentially eligible, examined for eligibility, confirmed eligible, included in the study, completing follow-up, and analysed*  All these data has been included in the results (page 4 – 6) |
|  |  | *(b) Give reasons for non-participation at each stage*  Done in page 4 |
|  |  | *(c) Consider use of a flow diagram*  Use of flow diagram was not necessary |
| Descriptive data | *14**** | *(a) Give characteristics of study participants (eg demographic, clinical, social) and information on exposures and potential confounders* |
|  |  | *(b) Indicate number of participants with missing data for each variable of interest*  All these data has been included in the results, in particular subsection “Socio-Demographic characteristics” (page 6) and Table 1 (page 7). |
| Outcome data | *15**** | *Report numbers of outcome events or summary measures*  Measures have been summarised in subheadings |
| Main results | *16* | *(a) Give unadjusted estimates and, if applicable, confounder-adjusted estimates and their precision (eg, 95% confidence interval). Make clear which confounders were adjusted for and why they were included* |
|  |  | *(b) Report category boundaries when continuous variables were categorized* |
|  |  | *(c) If relevant, consider translating estimates of relative risk into absolute risk for a meaningful time period*  All these data has been included in results and tables. Flow diagram was not necessary. Each main outcome was inserted in a subheading to assist for interpretation. |
| Other analyses | 17 | *Report other analyses done—eg analyses of subgroups and interactions, and sensitivity analyses*  All analysis has been reported in results section and in tables |
| Discussion | | |
| Key results | *18* | *Summarise key results with reference to study objectives*  Key results have been summarised (see page 6 – 11). Each objective of the study has been discussed in separate points respecting the order of introduction and results |
| Limitations | *19* | *Discuss limitations of the study, taking into account sources of potential bias or imprecision. Discuss both direction and magnitude of any potential bias*  Limitations have been discussed (Page 14) |
| Interpretation | *20* | *Give a cautious overall interpretation of results considering objectives, limitations, multiplicity of analyses, results from similar studies, and other relevant evidence*  A cautious interpretation considering other studies has been given for each specific aim. (page 12 – 14) |
| Generalisability | *21* | *Discuss the generalisability (external validity) of the study results*  Generalisability has been discussed at each specific point |
| Other information | | |
| Funding | 22 | *Give the source of funding and the role of the funders for the present study and, if applicable, for the original study on which the present article is based*  No funding was received |
