# Supplementary material for: Prevalence of maternal HIV infection and knowledge on mother–to–child transmission of HIV and its prevention among antenatal care attendees in a rural area in northwest Cameroon
Source: PLoS One. 2017 Feb 15;12(2):e0172102. doi: 10.1371/journal.pone.0172102 (PMC5310783; doi:10.1371/journal.pone.0172102)
Supplement: S2 File — (DOCX) [file pone.0172102.s002.docx]

**Prevalence of Maternal HIV Infection and Knowledge on Mother–To–Child Transmission of HIV and its Prevention among Antenatal Care Attendees in Babessi Subdivision**

**Data collection sheet**

**Socio–Demographic and obstetric characteristics**

1. Age: …………….
2. Marital status: 1. Married ….. 2. Single ….. 3. Divorced …….. 4. Widowed …… 5. Cohabitating ……
3. If married: 1. Monogamy ….. 2. Polygamy ……..
4. Religion: 1. Christian ….. 2. Muslim ….. 3. Traditionalist ….. 4. Others ……
5. Highest education attained: 1. None … 2. Primary …. 3. Secondary …. 4. Tertiary …
6. Occupation: 1. Housewife …. 2. Farmer …. 3. Unemployed ….. 4. Student ….. 5. Self–Employed ….. 6. Trader …. 7. Government work ….
7. Gestational age at start of fist ANC visit …………………………..
8. Gestational age of current pregnancy ………………………..
9. Gravid formula ………………………………
10. Number of ANC visits for the current pregnancy …………………

**Knowledge level of HIV / AIDS**

1. Have you ever heard about HIV/AIDS? 1. Yes …….. 2. No……..
2. If yes, how long have you been aware of HIV/AIDS? …………….
3. How does one get HIV/AIDS?

|  | No | Yes | I don’t know |
| --- | --- | --- | --- |
| Sexual intercourse |  |  |  |
| Mosquito bites |  |  |  |
| Blood transfusions |  |  |  |
| Infected instruments |  |  |  |
| Witchcraft |  |  |  |
| Physical contact (e.g hugging, handshake e.t.c) |  |  |  |

1. Can a healthy looking individual be infected with HIV/AIDS? 1. Yes ….. 2. No ….. 3. I don’t know …..
2. Can a woman infected with the HIV virus transmit it to her baby?

1. Yes ….. 2. No ….. 3. Don’t know …..

1. If yes to question 15, during which period can the HIV virus be transmitted to the baby?

|  | No | Yes | I don’t know |
| --- | --- | --- | --- |
| During pregnancy |  |  |  |
| During labour/delivery |  |  |  |
| During breastfeeding |  |  |  |

1. If yes to question 15, can transmission of HIV be prevented from an infected pregnant woman to her baby?
2. Yes ….. 2. No ….. 3. Don’t know …..
3. If yes to question 17, how can the transmission of HIV be prevented from an infected woman to her baby?

|  | No | Yes | I don’t know |
| --- | --- | --- | --- |
| Giving antiretroviral therapy to mother |  |  |  |
| Delivery by caesarean section. |  |  |  |
| Giving antiretroviral drugs to the newborn. |  |  |  |
| Avoid breastfeeding |  |  |  |
| Avoid mixed feeding |  |  |  |

**Permission of Spouse**

1. Do you need the permission of your husband or the father of your baby before engaging in HIV testing?

1. Yes ….. 2. No …..

1. How will your husband or father of your baby feel if you undergo HIV testing without his knowledge/permission/consent?

1. Angry ….. 2. Happy …… 3. Indifferent ….. 4. I don’t know …..

1. Do you know the HIV status of your husband/father of your baby?
2. Yes ….. 2. No …..

**Para clinical results**

1. Alere HIV test results: 1. Negative ………. 2. Positive ……………. 3. Indeterminate …………
2. If Alere is positive, results of SD Bioline: 1. Negative ………. 2. Positive ……
